# Genital Kaposi sarcoma in a HIV and syphilis co-infected patient: case presentation

**DOI:** 10.1186/s12879-019-4714-8

**Published:** 2019-12-30

**Authors:** Helena Lucia Barroso dos Reis, Dennis de Carvalho Ferreira, Neide Aparecida Tosato Boldrini, Carolina Galvão, João Victor Jacomele Caldas, Marize Freitas Santos Neves, Philippe Godefroy

**Affiliations:** 1 Maternal and Child Health by Fluminense Federal University, Niteroi, Brazil; 2 Post-graduation Program in Infectious Diseases at the Federal University of Espírito Santo, (UFES/ES), Vitoria, Brazil; 3 Oral Pathology by Federal University of Rio de Janeiro, (UFRJ/RJ), Rio de Janeiro, Rio de Janeiro, Brazil; 4 Adjunt Professor at School of Dentistry, Veiga de Almeida University (UVA) and Estácio de Sá University, Rio de Janeiro, Brazil; 5 Infectious Diseases by Federal University of Espírito Santo, (UFES/ES), Vitória, Vitoria, ES Brazil; 6 Gynecology and Obstetrics Professor at Federal University of Espírito Santo, Vitoria, Brazil; 7 Nurse by Anhanguera University, RJ, Coordinator at the Municipal Matemity Alzira Reis Vieira Ferreira Niterói, Rio de Janeiro, Brazil; 8 Vila Velha University (UVV-ES) - Vila Velha ES,Brazil, Vitoria, Brazil; 9 Gynecology and Obstetrics at The Federal University of Espírito Santo, (UFES/ES), Vitoria, Brazil; 10 Gynecology and Obstetrics Specialist by FEBRASGO and Gynecology and Obstetrics Professor at Federal University of Espírito Santo, Vitoria, Brazil; 11 Maternal and Child Health by Fluminense Federal University, (UFF/RJ), Niteroi, Brazil; 12 Medical Coordinator in The Intermedial Notredame Group, Rio de Janeiro, Brazil

**Keywords:** Sarcoma, Kaposi, HIV, Syphilis

## Abstract

**Background:**

Kaposi sarcoma, as an epidemiological factor, is associated with acquired immunodeficiency syndrome (AIDS) and it is related to human herpes virus (HHV-8), as well as a higher prevalence in males and non-genital involvement. Vulvar localization is quite infrequent; therefore it may be considered in the differential diagnosis of genital lesions, especially in HIV patients.

**Case presentation:**

We describe the atypical presentation of a female HIV patient with multiple comorbidities, with the clinical manifestation of Kaposi sarcoma (KS) in a vulvar region that was initially diagnosed as a syphilitic gumma. The patient underwent a biopsy of the lesion, and histopathology revealed a Kaposi sarcoma.

**Discussion:**

This case reinforces that the pathogenesis of Kaposi sarcoma is still unclear and that probably multiple factors, regarding both the virus and the patient characteristics may lead to carcinogenesis. Conclusion: It is imperative to seek more excellent knowledge about this disease, to facilitate the diagnosis, to warrant the appropriate treatment and to improve the prognosis of the patient, especially the genital lesions.

## Background

Human immunodeficiency virus (HIV) infection is a severe global public health problem, and since Kaposi sarcoma (KS) is the most common neoplasm among HIV infected patients, it is becoming increasingly crucial to study clinical manifestations, diagnosis, evolution, and treatment of this neoplasia [1]. KS is a vascular neoplasm whose appearance in an HIV-infected patient is related to herpesvirus 8 (HHV- 8) [2]. This systemic disease has already shown to have an extensive pleomorphic presentation, a fact that many professionals are unaware of in clinical practice, only recognizing its most common manifestation, a purplish skin lesion usually on the face or the upper part of the thorax. KS is more common in men, and the onset in a female patient, especially in the genital region, may be misdiagnosed due to the similarity to non-malignant diseases such as genital infection like syphilis and genital herpes [3]. The knowledge and analysis of patient with genital Kaposi Sarcoma and Aids is a challenge for the health professional to diagnose and treat those diseases due to the presence of opportunistic diseases and other sexually transmitted diseases [4].

## Case presentation

**Fig. 1:**
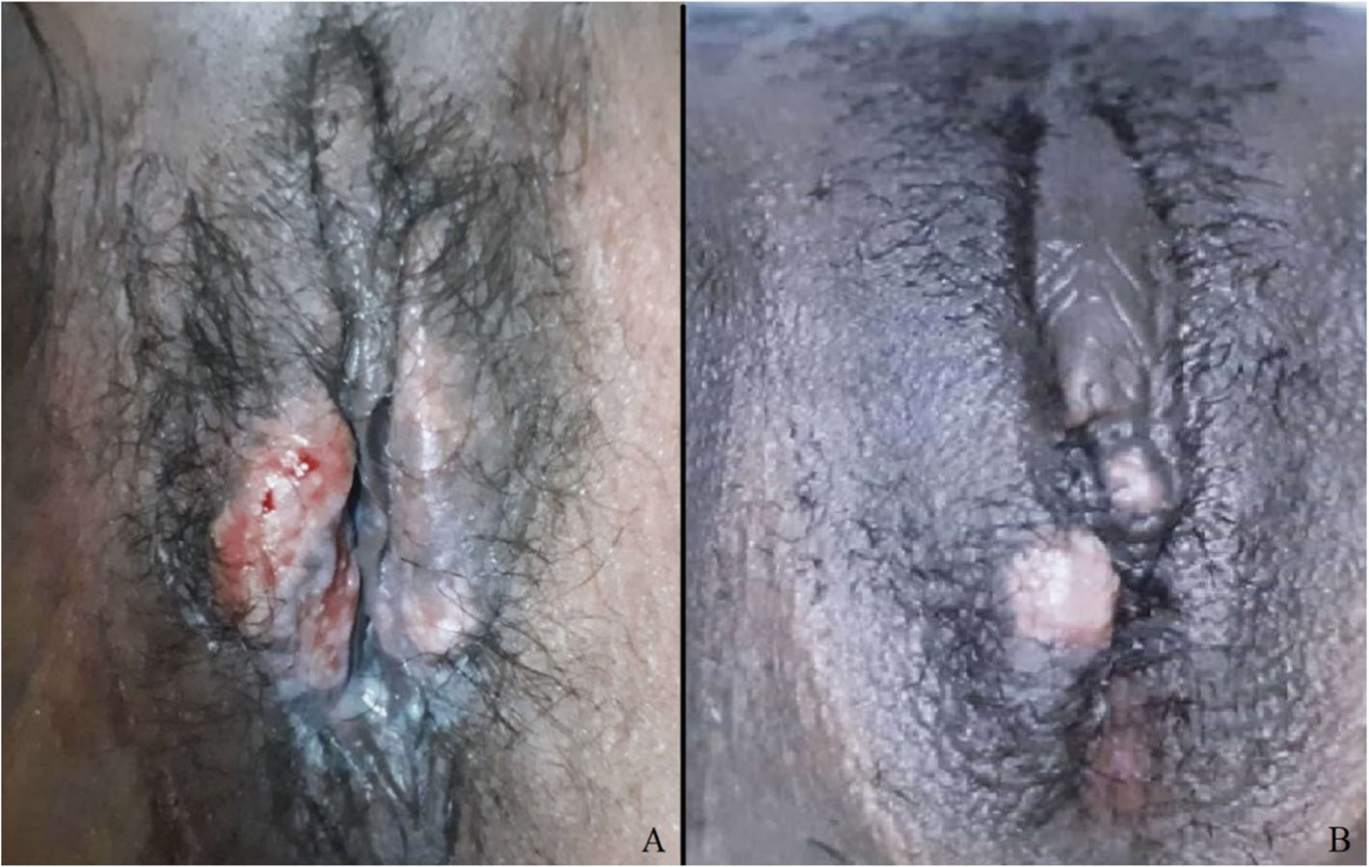
**a** Multiple ulcers on right labium minor and majus suggestive of Syphilis **b** A persistent nodular lesion in the vulvar’s right labium majus: Kaposi sarcoma

A 33-year-old black female patient, housewife, born and resident in the city of Niteroi, Brazil attended a Municipal Hospital with complaints of fever, weakness, prostration, chest pain, non-productive cough, long-term dysuria, and significant weight loss of 20 kg in the last 6 months. At the time of the care, a rapid test for HIV was positive. She reported that approximately six months before, she noticed the appearance of vaginal discharge and genital ulcers, and in the previous three months, with afternoon fever along with all other complaints described above. In relation to the vital signs, she presented blood pressure of 100 × 40 mmHg, heart rate of 104 beats per minute, respiratory rate of 24 breaths per minute and axillary temperature of 36.2 °C. Furthermore, the patient was pale (++ / 4+), anicteric, non-cyanotic, eupneic, very emaciated, oriented but with slowed reasoning, responsive to the verbal request. She had exuberant oral candidiasis, angular cheilitis, and purulent crusts on her lips. Gynecological examination showed ulcers on right labium minor and majus, presence of vaginal discharge, and nodular lesion on the right labium majus. Moreover, she presented umbilicated pubic lesions indicative of Molluscum contagiosum. Upon laboratory tests, she presented significant pancytopenia and altered liver function enzymes. The other biochemical blood tests were normal. Sputum examination of an acid-alcohol resistant bacillus (BAAR) was positive, and chest X-ray revealed bilateral infiltrations, being more pronounced in the two lower thirds. Abdominal ultrasound scan presented liver with increased dimensions suggesting steatosis. Retroperitoneal lymph node enlargement was described, with the largest lymph node measuring 2.2 cm and mild/moderate ascites. The pelvic endovaginal ultrasound was unremarkable. A Rifampin, Isoniazid, Pyrazinamide, and Ethambutol (RIPE) therapy scheme for tuberculosis was started, a red blood cell transfusion for the correction of anemia, antiretroviral therapy, Sulfamethoxazole-trimethoprim for urinary tract infection and Acyclovir for the initial genital herpes lesion suspicion. A Venereal Disease Research Laboratory (VDRL) non-treponemal serum test showed a 1:8 result. Also a cerebrospinal fluid VDRL was performed with a negative result. Thus, therapy with crystalline Penicillin G was started for 21 days. Material was collected from the vulvar lesion to perform a Dark Field Examination, which evidenced live treponema. After the 17th day of penicillin, a biopsy of a persistent nodular lesion was performed in the external surface of the right labium majus (Fig. 1b), whose microscopic analysis of the obtained material showed proliferation of endothelial cells and the presence of fusiform cells, as well as vascular neoformation and extravasated red blood cells, confirming the diagnosis of Kaposi’s sarcoma. Also, an excisional biopsy of shiny and umbilicated pubic lesions was performed confirming the Molluscum contagiosum clinical diagnosis. The antiretroviral regimen used was Lamivudine, Stavudine, and Efavirenz, and the laboratory results after the treatment were: the viral load of 170 copies, log 2230; TCD4 count 314; TCD8 count 336 and TCD4 / TCD8 ratio equal to 0.93. The treatment employed contributed to the patient clinical and immunological improvement whose oral lesions regressed completely after the improvement of the patient’s immunity into two years.

## Discussion and conclusions

The etiopathogenesis of KS is not entirely elucidated but is known to include exposure to an infectious agent in addition to HIV [5]. The development of KS is related to HIV infection and, especially, to opportunistic infections that may lead to the inflammatory response to such diseases [6]. Among the infectious agents that contribute to the development of KS, HHV- 8, which is found in more than 90% of KS lesions in individuals with AIDS [2, 4–7], stands out. However, HIV infection is not directly related to the development of KS, since it has never been possible to isolate it in neoplastic tissue [8]. The indirect contribution of HIV lies in the depletion of CD4 lymphocytes and the stimulation of excessive production of inflammatory mediators [9–11]. Because of the pathogenesis, as mentioned above, the development of this neoplasia may be related to HIV infection. The patient had HIV and HHV-8 and had several concomitant opportunistic infections such as syphilis, which may present aggressive or atypical clinical manifestation, as well as, pulmonary tuberculosis, oral candidiasis and Molluscum contagiosum. Thus, immunosuppression caused by HIV allowed the development of these multiple infections, which in turn triggered inflammatory responses with excessive production of lymphokines and cytokines, which eventually contributed to the development of KS [6]. The co-existence of syphilis and KS in the genital area of this HIV patient caused a difficult treatment challenge to treat both diseases adequately. In order to avoid neurological problems the early diagnosis of syphilis is imperative [10]. The knowledge and analysis of cases, as reported, still draw attention to the overlap of opportunistic diseases and other sexually transmitted diseases [2, 11]. Overall, KS restricted to the external genitalia is an infrequent finding that is more common in men, particularly on the penis. Meanwhile, it is very uncommon in women [12]. Since KS is the most prevalent neoplasm among HIV patients and in the face of an HIV / AIDS pandemic, it is imperative to seek more excellent knowledge about this disease, in order to facilitate the diagnosis process and to warrant the appropriate treatment in order to improve the prognosis of the patient especially the genital lesions that must be biopsied for diagnosis.

## Data Availability

All data generated or analyzed during this study are included in this published article.

## Abbreviations

AIDS: Acquired immunodeficiency syndrome
BAAR: Acid-alcohol resistant bacillus
HHV- 8: human herpesvirus 8
HIV: Human immunodeficiency virus
KS: Kaposi sarcoma
RIPE: Rifampin, Isoniazid, Pyrazinamide, and Ethambutol
TCD4: T-lymphocytes CD4
TCD8: T-lymphocytes CD8
VDRL: Venereal Disease Research Laboratory
